# Supplementary figures and images for: Visual enumeration remains challenging for multimodal generative AI
Source: PLoS One. 2025 Sep 12;20(9):e0331566. doi: 10.1371/journal.pone.0331566 (PMC12431670; doi:10.1371/journal.pone.0331566)

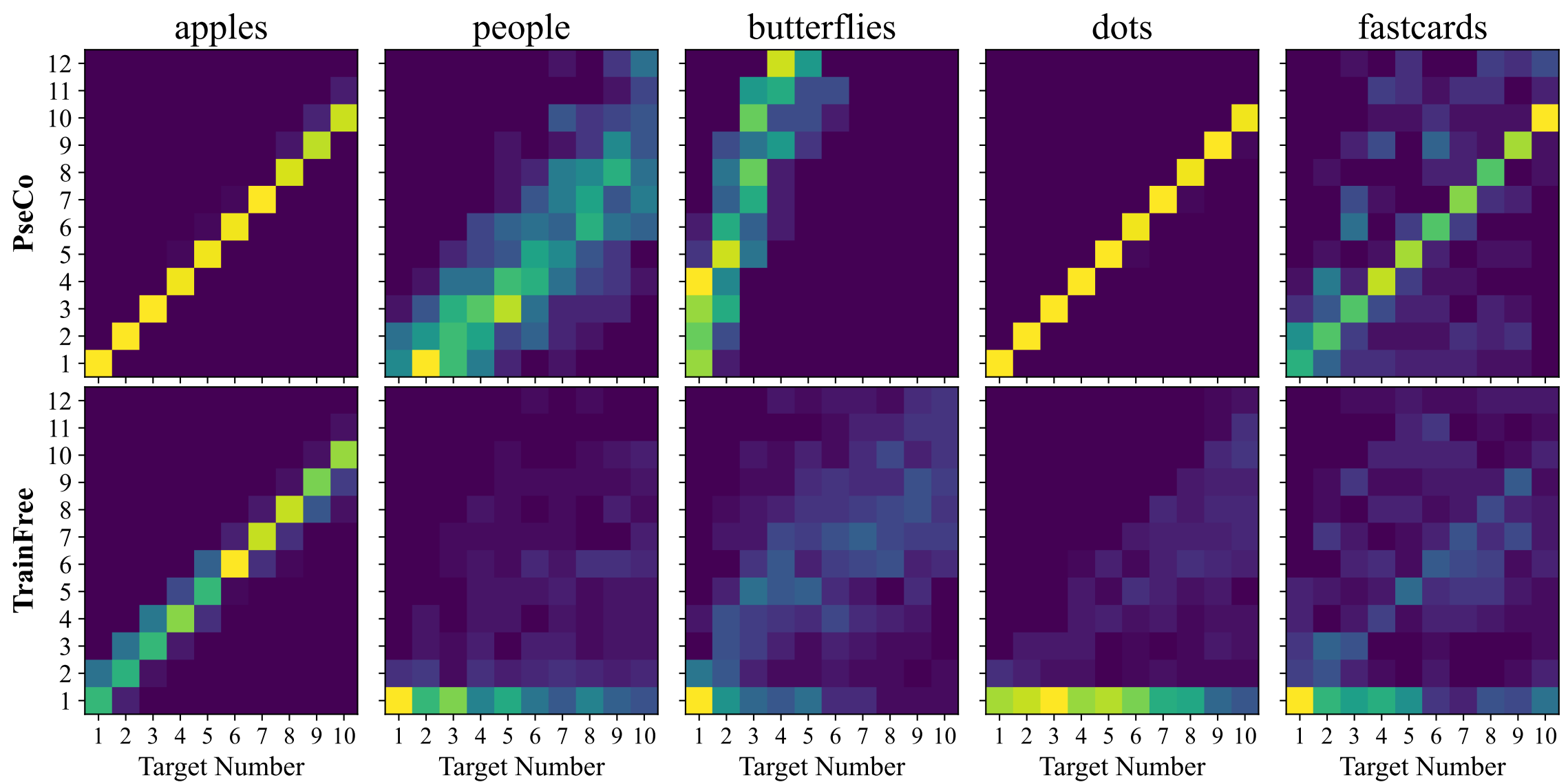

Supplement: S2 Fig — Each panel shows the distribution of models’ responses across different object categories: apples, people, butterflies, dots and fast cards. The x-axis represents the target number, while the y-axis represents the corresponding model responses. Response frequency is encoded using a perceptually uniform colormap (blue = 0%, yellow = 100%). (PDF) [file pone.0331566.s002.pdf]

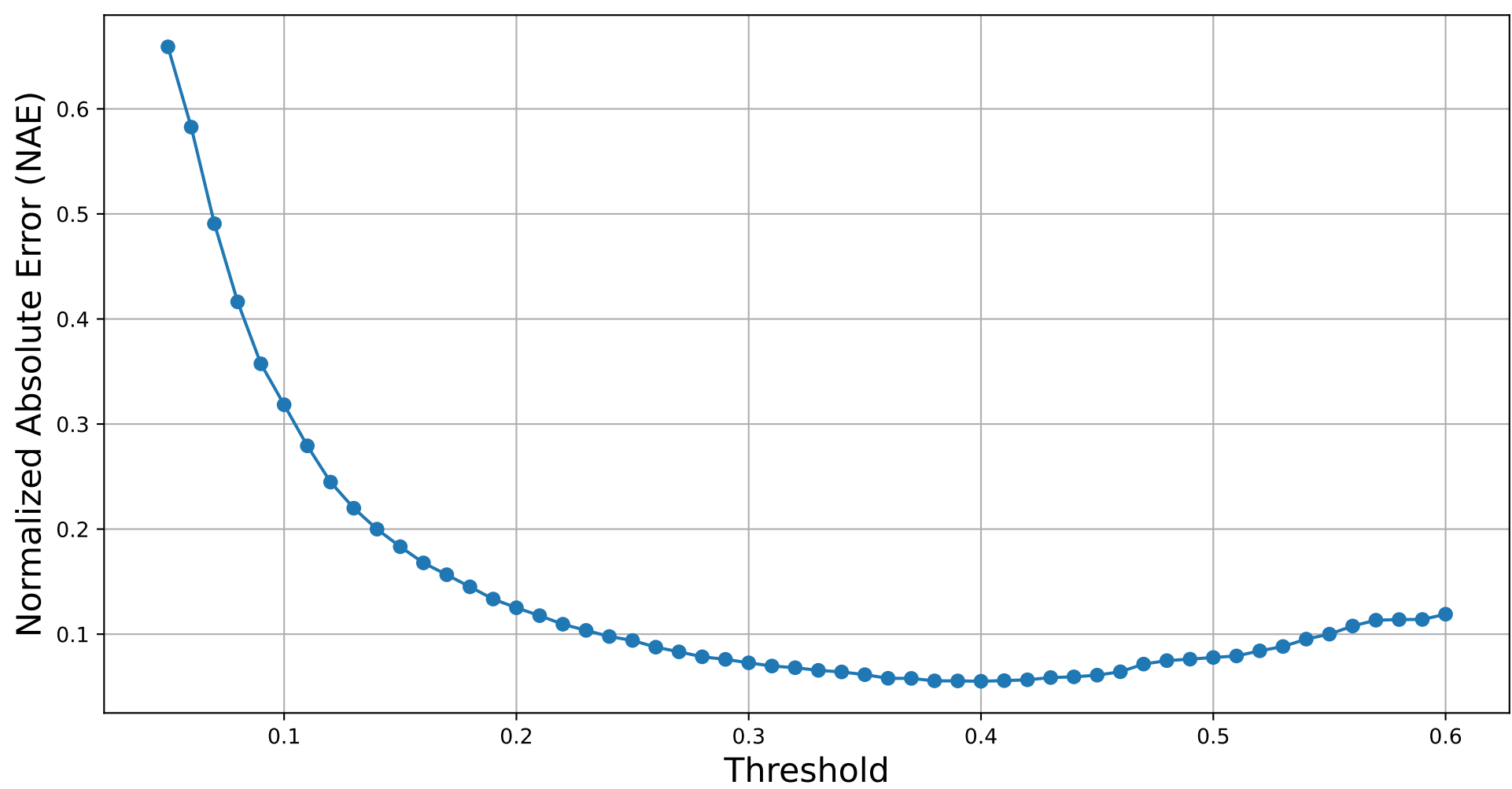

Supplement: S4 Fig — The optimal threshold was found at 0.40. (PDF) [file pone.0331566.s004.pdf]
