# Supplementary material for: Visual enumeration remains challenging for multimodal generative AI
Source: PLoS One. 2025 Sep 12;20(9):e0331566. doi: 10.1371/journal.pone.0331566 (PMC12431670; doi:10.1371/journal.pone.0331566)
Supplement: S3 Fig — The numerical information in the text is highlighted in red. (PDF) [file pone.0331566.s003.pdf]

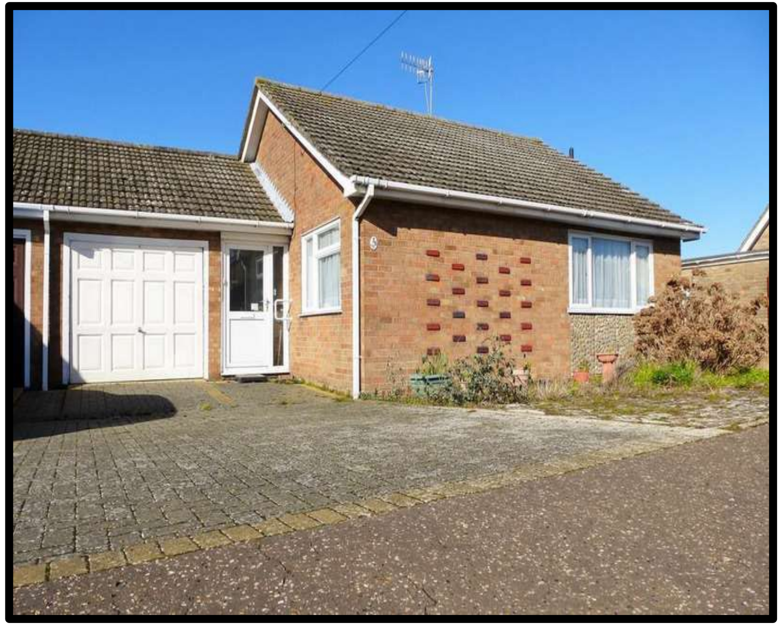

3 Bedrooms Semi Detached Bungalow for sale in Cromer

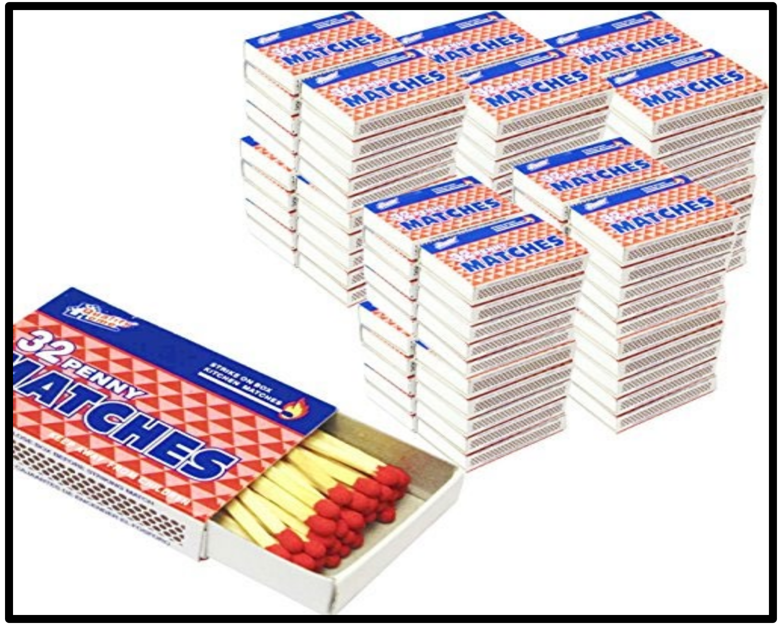

100 Packs Matches 32 Count Strike on Box Kitchen Camping Fire Wholesale Lot Bulk]()

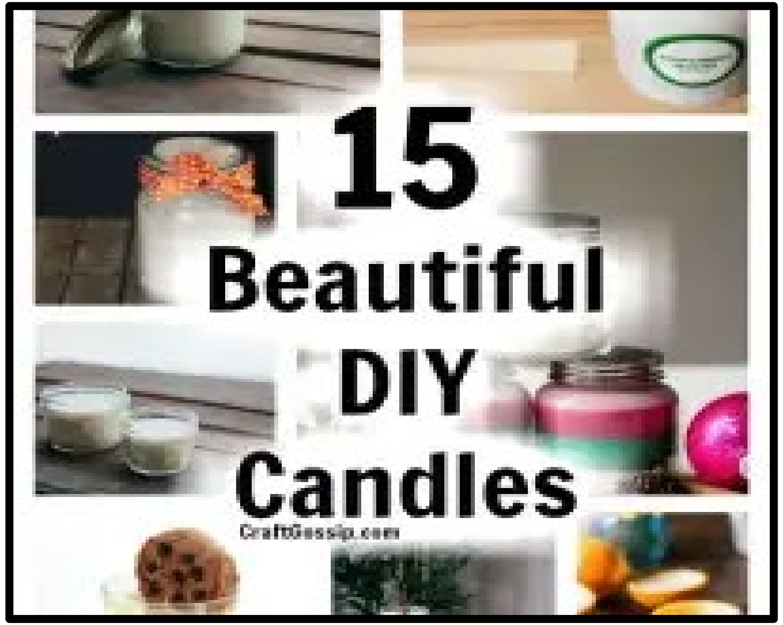

15 Beautiful DIY Candles
