# Supplementary material for: Visual enumeration remains challenging for multimodal generative AI
Source: PLoS One. 2025 Sep 12;20(9):e0331566. doi: 10.1371/journal.pone.0331566 (PMC12431670; doi:10.1371/journal.pone.0331566)
Supplement: S1 Fig — On the left is the random choice model, while on the right is the ideal human observer, which has perfect accuracy in the subitizing range (1-4) and approximately estimates larger numbers according to Weber’s law. (PDF) [file pone.0331566.s001.pdf]

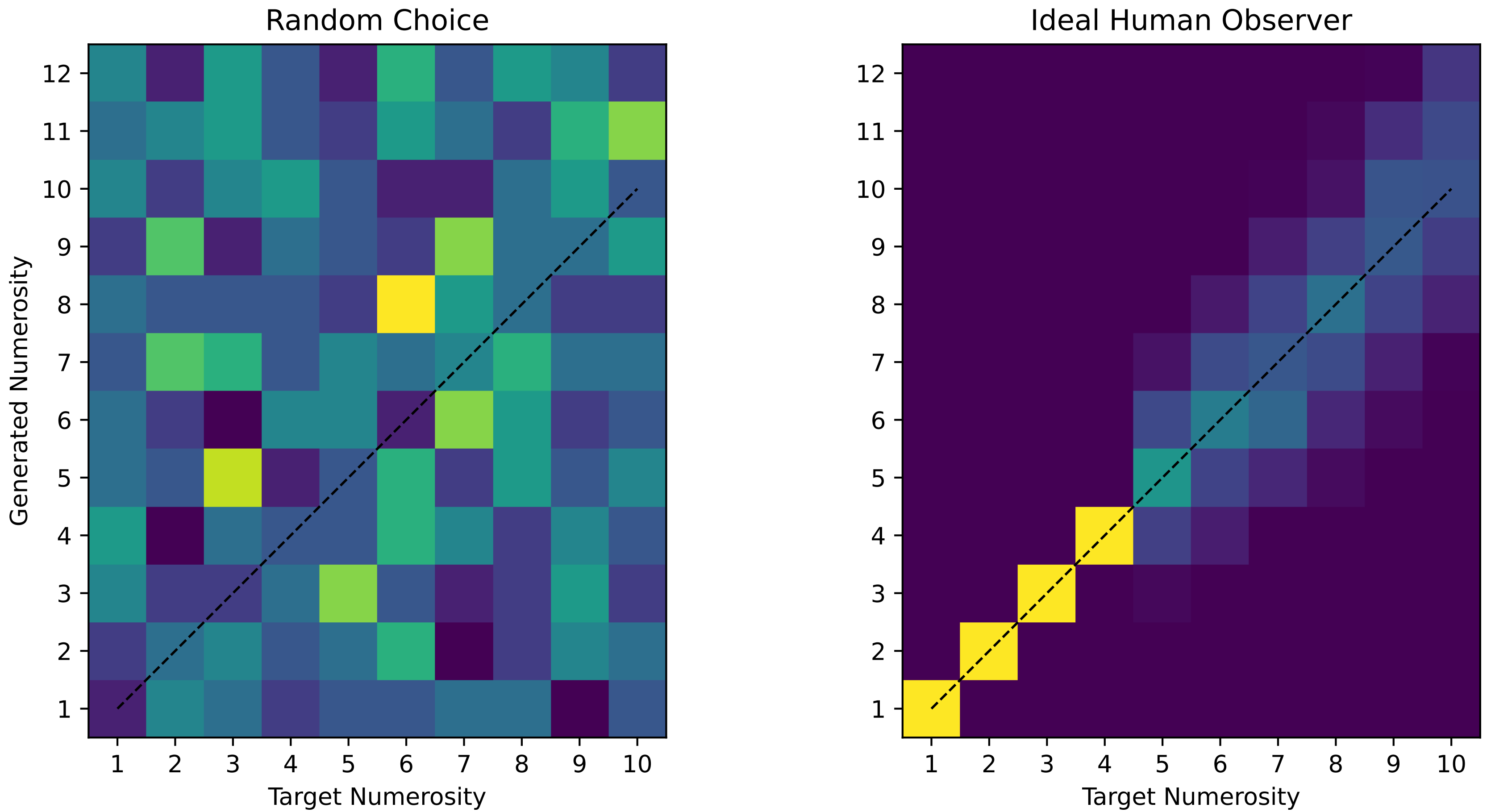

Random Choice
Ideal Human Observer
Generated Numerosity
Target Numerosity
Target Numerosity
1
2
3
4
5
6
7
8
9
10
11
12
